# Prediction of the Mechanism of Shaoyao Gancao Decoction in the Treatment of Alopecia Areata by Network Pharmacology and Its Preliminary Verification Study

**DOI:** 10.1155/2022/5764107

**Published:** 2022-04-07

**Authors:** Shuying Lv, Lei Wang, Yuhang Duan, Dan Huang, Dingquan Yang

**Affiliations:** ^1^School of Clinical Medicine, Beijing University of Chinese Medicine, Beijing 100029, China; ^2^Department of Dermatology, China-Japan Friendship Hospital, Beijing 100029, China; ^3^First Affiliated Hospital of Guangzhou University of Chinese Medicine, Guangzhou 510006, China

## Abstract

**Objective:**

To explore the mechanism of Shaoyao Gancao decoction (SGD) in treatment of alopecia areata (AA) by network pharmacology and animal experiments.

**Methods:**

Based on the traditional Chinese medicine systems pharmacology database and analysis platform (TCMSP), the components and targets of SGD were determined. Then, the related targets of AA were retrieved from DrugBank, GeneCards, OMIM, and DisGeNET databases. The intersection of drug targets and disease targets was determined, and the key targets of the protein-protein interaction network were obtained with the String database. Gene Ontology (GO) biological process enrichment analysis and Kyoto Encyclopedia of Genes and Genomes (KEGG) pathway enrichment analysis of potential key targets were carried out using the DAVID database using AutoDock for molecular docking verification. Finally, the key pathway was validated by animal experiments.

**Results:**

A total of 102 active components, 212 predicted targets, and 812 AA disease-related targets were obtained. Topological analysis yielded 45 key targets of SGD in the treatment of AA, including IL-6, PTGS2, TNF, VEGFA, CCL2, IL-1B, CXCL8, CASP3, MPO, and IL-10. There were 324 GO entries obtained through GO biological process enrichment analysis, and 20 pathways were obtained through KEGG pathway enrichment analysis, involving the PI3K-Akt signaling pathway, osteoclast differentiation, and Jak-STAT signaling pathway. The molecular docking results showed effective ingredients (quercetin, kaempferol, and 7-methoxy-2-methyl isoflavone) have good docking results with targets (IL-6, PTGS2, and TNF). The results of animal experiments showed that SGD can effectively upregulate the expression of PI3K and AKT proteins.

**Conclusion:**

This is the first in-depth study on the mechanism of SGD's treatment effect in AA using network pharmacology, and preliminary animal experiments verified that it is closely related to the PI3K/AKT signaling pathway. This finding may provide a new basis for SGD's clinical application in AA.

## 1. Introduction

Alopecia areata (AA) is a T-cell-mediated nonscarring alopecia characterized by an autoimmune reaction in the hair follicle. AA is associated with various factors such as genetics, neurological and psychiatric conditions, oxidative stress, and viral infections. Studies have shown that the lifetime prevalence of AA is about 2% globally and 0.27% in China [1]. AA is a disfiguring skin disease that often has a negative impact on patients' psychological health and quality of life. In terms of treatment, both glucocorticoids and immunosuppression are recommended by *The Chinese Guidelines for the Treatment of Alopecia Areata (2019)* [2], but all have the limitation of offering mainly symptom relief and not eradicating the disease; these treatments are often accompanied by various adverse effects and relapse after discontinuation. Therefore, it is crucial to find new, more durable, and effective treatments for AA.

Shaoyao Gancao decoction (SGD) is derived from Zhang Zhongjing's *Treatise on Febrile and Miscellaneous Diseases*. The whole formula consists of two herbs: Shaoyao and Gancao. Shaoyao is sour in taste and cold in property, with the effect of nourishing blood and astringing *yin*, softening the liver and relieving pain; Gancao is sweet in taste and warm in property, strengthening the spleen and benefiting *qi* and relieving pain. The combination of the two herbs nourishes the yin with sour and sweet and harmonizes the liver and spleen as well as relieves pain. Modern pharmacological studies [3] have shown that SGD has analgesic and anti-inflammatory effects, which are commonly used clinically for the treatment of related painful and inflammatory diseases. A clinical study [4] has confirmed the safe and effective treatment of AA in adults and children with total glucosides of paeony capsules containing Shaoyao components. In addition, our group's previous study has shown that Shaoyao Gancao granule (a traditional Chinese medicine prescription made from Shaoyao and Gancao) is safe and effective in the clinical treatment of severe AA and can regulate the expression of Th1/Th2, Th17/Treg cells, and their related factors, but the specific mechanism of action is unclear [5].

Network pharmacology is an emerging systems biology research method that combines modern medicine and bioinformatics. This method uses biological databases to construct and demonstrate the relationships among diseases, targets, and drugs, thus systematically identifying the possible mechanisms of action of drugs [6]. The characteristics of this approach are consistent with the multicomponent, multitarget, and multipathway action characteristics of traditional Chinese medicine (TCM).

In this study, we investigated active components of SGD through network pharmacology, constructed a multilevel bioinformatics network of SGD for AA treatment, and successfully predicted the potential key targets and pathways of SGD for the treatment of AA, which strongly reflected the multicomponent, multitarget, and multipathway action characteristics of SGD. At the same time, molecular docking and animal experiments were used to validate the pharmacological mechanism, providing a theoretical basis for further research.

## 2. Materials and Methods

### 2.1. Preparation of SGD

The SGD granules formula consisted of 45 g of Shaoyao and 15 g of Gancao, prepared by the Pharmacy Department of the China-Japan Friendship Hospital, 10 g per bag (containing 30 g of raw medicine), in which the content of paeoniflorin was not less than 900 mg and that of glycyrrhetinic acid was not less than 75 mg.

### 2.2. Animals and Experimental Groups

We obtained 30 female C57BL/6J mice (22 ± 3 g), 6∼8 weeks old, from Beijing Vital River Laboratory Animal Technology Co., Ltd. (Beijing, China, license no. SCXK, 2016-0006). All the animal experiments strictly followed the relevant standards and requirements of the animal experimental platform of the Institute of Clinical Medicine, China-Japan Friendship Hospital.

The 30 mice were randomly and equally divided into three groups: the control group, the model group, and the SGD group. The latter two groups were treated with topical imiquimod cream combined with 21-day chronic unpredictable mild stress to establish a mouse model of AA, after which the SGD group was given SGD (9.76 g/kg) by gavage for 21 consecutive days.

### 2.3. Reagents

Reagents included imiquimod cream (Sichuan Mingxin Pharmaceutical Company), phosphoinositide 3-kinase (PI3K) P85 alpha monoclonal antibody (60225-1-lg, Proteintech), AKT (60203-2-lg, Proteintech), goat-anti-mouse IgG-HRP (115-035-003, Jackson ImmunoResearch), and RIPA-containing lysis buffer (#9806, Cell Signaling Technology).

### 2.4. Instruments

We used a cryogenic frozen centrifuge (ThermoFisher, ID: Fresco17), Bio-Rad Mini PROTEAN Tetra, Bio-Rad Mini Trans-Blot, Bio-Rad PowerPac Basic.

### 2.5. Network Pharmacology-Based Mechanism for Predicting Effect of SGD on AA

#### 2.5.1. Screening of Therapeutic Targets of Active Components Related to SGD

The chemical components of Shaoyao and Gancao were researched separately in the traditional Chinese medicine systems pharmacology database and analysis platform (TCMSP; https://lsp.nwu.edu.cn/tcmsp.php). The screening criteria for the main chemical components were set as oral bioavailability (OB) ≥ 30% and drug-like index (DL) ≥ 0.18. The Universal Protein Database (UniProt, https://www.uniprot.org/) was then used to standardize the target information.

#### 2.5.2. Screening of Alopecia Areata Gene Targets

The keywords “alopecia areata” were used to screen for AA-related gene targets in the DrugBank database (https://www.drugbank.ca/), GeneCards database (https://www.genecards.org, Version 4.11.0), Online Mendelian Inheritance in Man (OMIM) (https://omim.org/), and DisGeNET database (https://www.disgenet.org/). The disease targets obtained from these databases were pooled and duplicate targets were removed to obtain AA disease-related targets.

#### 2.5.3. Construction and Analysis of SGD Herbs-Compounds-Targets (H-C-T) Network

The potential target genes of SGD in the treatment of AA were the intersecting parts of the target genes corresponding to the action of active components of SGD and the target genes related to AA. The overlapping partial genes were visualized by Venn diagrams (https://bioinfogp.cnb.csic.es/tools/venny/). Cytoscape 3.8.0 software was then used to construct the network of interactions between overlapping genes, bioactive compounds, and herbs, called the H-C-T network.

#### 2.5.4. Construction of Core Targets' Protein-Protein Interaction Network

The potential targets of SGD for AA treatment were imported into the STRING 11.0 database (https://string-db.org/), setting the species genus as “*Homo sapiens*,” the minimum interaction score as “medium confidence (0.400),” and hiding the free sites. The protein-protein interaction (PPI) network of SGD for AA was obtained using Cytoscape 3.8.0 software for network topology analysis.

#### 2.5.5. Enrichment Analysis of Core Targets

Gene Ontology (GO) biological process enrichment analysis and Kyoto Encyclopedia of Genes and Genomes (KEGG) pathway enrichment analysis were performed on the core targets for the action of SGD using the David database (https://david.ncifcrf.gov/).

#### 2.5.6. Molecular Docking

We screened the highest five-node degree active components in the H-C-T network of SGD and the highest five-node degree core proteins in the PPI network. Then, the potential interaction of ligands in the complex molecular network was studied by molecular docking simulation. The 3D structure Protein Data Bank (PDB) format files of the target proteins were retrieved and downloaded from the database (https://www.rcsb.org/). Subsequently, AutoDock Vina was used to verify molecular docking between core components and core targets. The molecular docking results were optimized and mapped with the help of PyMOL software.

### 2.6. Validation of the Pharmacological Effect of SGD on AA by Western Blot Analysis

Mouse skin tissues were homogenized in RIPA-containing lysis buffer and centrifuged, and protein concentrations were determined. Total proteins were separated by electrophoresis on an 8% separation gel and electrotransferred to a 0.2 mm pore size NC membrane. After being incubated with 5% nonfat dry milk in Tris-buffered saline for 2 h at room temperature, the membranes were incubated with antibodies at 4°C overnight. Primary antibodies against AKT (60203-2-lg) and PI3K (60225-1-lg) were purchased from Proteintech (Wuhan, China). After washing three times with Tris-buffered saline with Tween 20 (TBST), the membranes were incubated with goat-anti-mouse secondary antibody (1 : 10,000), washed as previously described, and then detected by western blotting. The grayscale intensity of the protein bands was analyzed and quantified using Image Lab.

### 2.7. Statistical Analysis

SPSS 21.0 was used for the analysis. Normally distributed data were expressed as mean ± standard deviation, nonnormal distributions were expressed as median and quartiles, and count data were expressed as percentages. When comparing the sample means of two groups, the independent samples *t*-test was used for normally distributed data, the rank sum (Kruskal–Wallis H) test was used for nonnormally distributed data, and the chi-square test was used for count data. Differences were considered statistically significant when *P* < 0.05.

## 3. Results

### 3.1. Screening Results of Chemical Components of SGD

A total of 105 active ingredients were screened by searching the TCMSP database, including 13 Shaoyao roots and 92 Gancao roots, among which three compounds (MOL000211, MOL000359, and MOL000422) were repeated. After deduplication, 102 active ingredients were obtained. The results are shown in Table 1.

### 3.2. Construction of the SGD H-C-T Network

**Figure 1:**
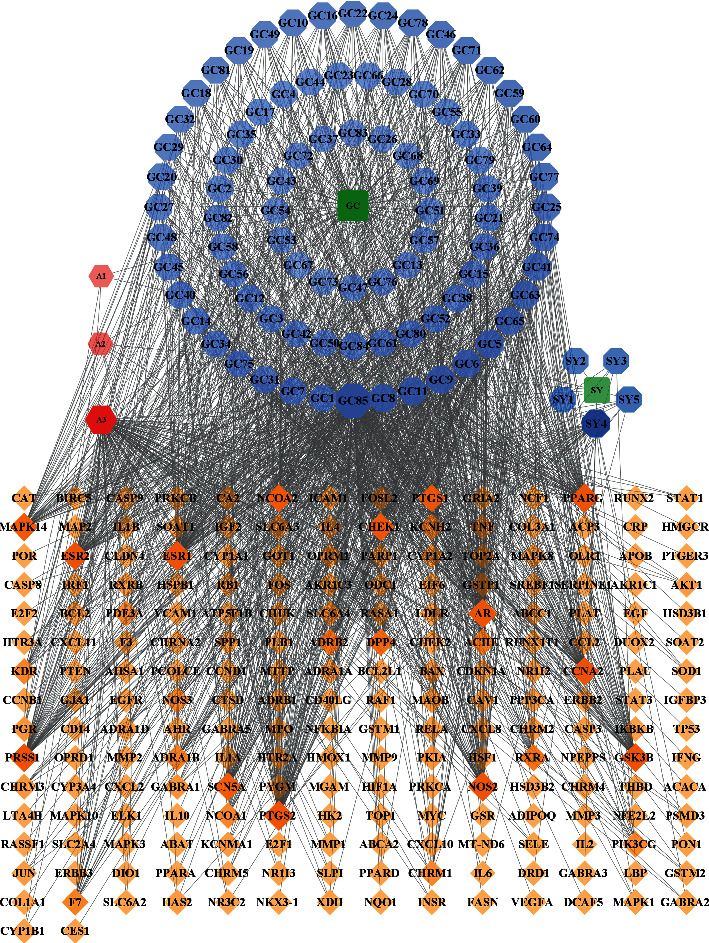
Shaoyao Gancao decoction (SGD) herbs, compounds, targets (H-C-T) network. Blue hexagons at the top represent active components of Gancao; blue hexagons on the upper right represent active components of Shaoyao; red hexagons represent active components shared by both Shaoyao and Gancao; orange squares represent target genes; and green squares represent drugs.

Among the abovementioned 102 related active compounds of SGD, nine compounds (MOL001910, MOL001921, MOL001925, MOL001928, MOL001930, MOL004860, MOL004905, MOL004917, and MOL005013) failed to match relevant targets. The remaining 93 components were matched to 1447 human gene targets in the TCMSP database, and 212 components were finally obtained after deduplication. By importing active compounds and corresponding targets into Cytoscape 3.8.0 software, the network of interactions between herbs, compounds, and target genes was constructed, with 307 nodes and 1543 edges, as shown in Figure 1.

### 3.3. AA Disease Target Genes

**Figure 2:**
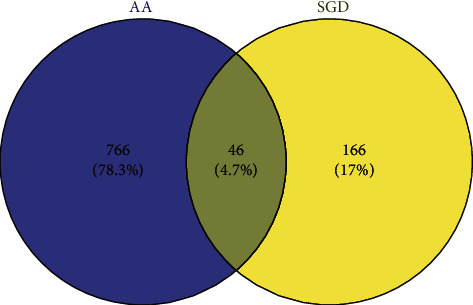
Venn diagram of Shaoyao Dancao decoction (SGD) and alopecia areata (AA) common targets.

The keywords “alopecia areata” were searched in the DrugBank, GeneCards, OMIM, and DisGeNET databases. After deduplication, 812 AA disease target genes were obtained. The resulting Venn diagram of the intersection of SGD main active compounds' target genes and AA target genes is shown in Figure 2.

### 3.4. Construction and Analysis of the PPI Network

**Figure 3:**
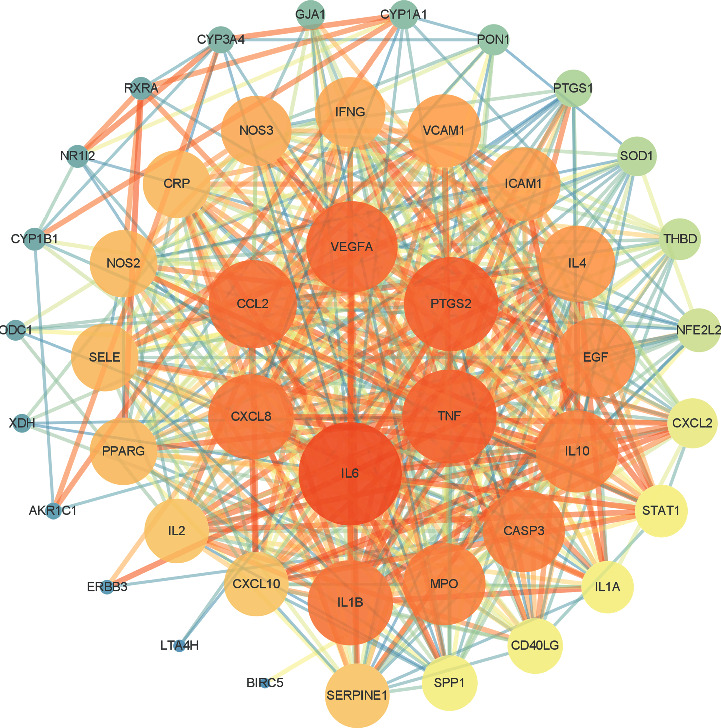
Protein-protein interaction (PPI) network diagram of key targets. Node size is positively correlated with degree, and node color changing from blue to orange corresponds to an increasing degree; the thickness of the edge is positively correlated with the binding fraction between proteins.

The PPI network (Figure 3) was constructed by importing 46 intersecting target genes into the STRING database, with 46 nodes and 465 edges (one target was not connected to other proteins, so it was not shown in PPI). The details of the 45 potential targets are listed in Table 2. The top 10 genes with degree values were IL-6, PTGS2, TNF, VEGFA, CCL2, IL-1B, CXCL8, CASP3, MPO, and IL-10. The abovementioned target genes are closely linked to other target genes and may play an important role in the treatment of AA.

### 3.5. GO and KEGG Enrichment Analysis Results

**Figure 4:**
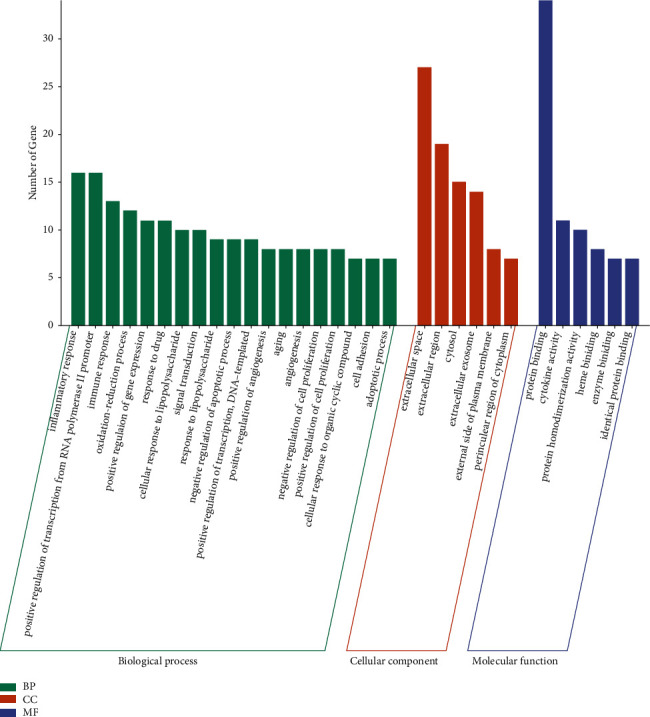
Gene ontology (GO) enrichment biological process map.

Through GO biological process enrichment analysis, 45 core targets yielded a total of 324 GO entries using David, including 270 biological process (BP) entries, 35 molecular function (MF) entries, and 19 cell composition (CC) entries, as shown in Figure 4.

**Figure 5:**
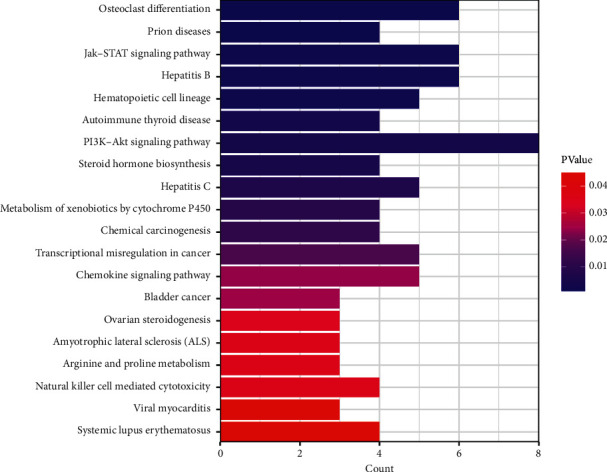
Kyoto Encyclopedia of Genes and Genomes (KEGG) pathway enrichment map.

Through the KEGG pathway enrichment analysis, 45 potential targets were mapped to a total of 60 signaling pathways using David, 20 of which were identified as *P* < 0.05, as shown in Figure 5.

### 3.6. Molecular Docking Results

**Figure 6:**
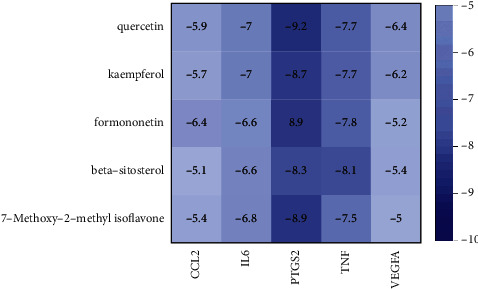
Heat map of core active components and core targets' binding energy.

**Figure 7:**
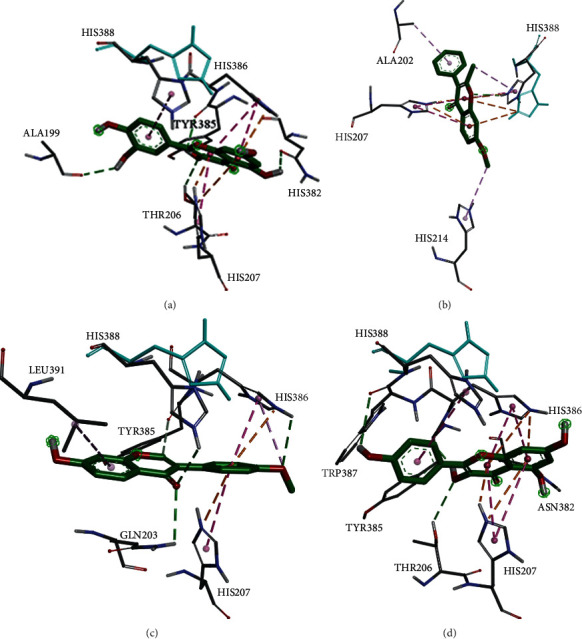
Molecular docking pattern of core active components and core targets. The four docking pattern diagrams representing higher binding energy are (a) PTGS2-quercetin, (b) PTGS2-7-methoxy-2-methyl isoflavone, (c) PTGS2- formononetin, and (d) PTGS2- kaempferol.

Quercetin, kaempferol, 7-methoxy-2-methyl isoflavone, beta-sitosterol, and formononetin were molecularly docked with IL-6, PTGS2, TNF, VEGFA, and CCL2. The lower the binding energy, the better the docking results. The results showed that the binding energy of each component to the target protein was less than −5 kcal/mol, among which quercetin had the best combination with PTGS2 (−9.2 kcal/mol), as shown in Figure 6. The molecular docking simulation diagram is shown in Figure 7.

### 3.7. Validation of Potential Therapeutic Mechanism of SGD by Western Blot

The AKT protein level of mice in the model group was 0.99 ± 0.22, and the result was lower compared with that of the control group of 1.89 ± 0.39 (*P*=0.021). The AKT protein level in mice in the SGD group was 1.92 ± 0.45, and the result was higher compared with the model group (*P*=0.021). The AKT protein levels of mice in the control and SGD groups were similar, with no significant statistical difference (*P*=0.773).

**Figure 8:**
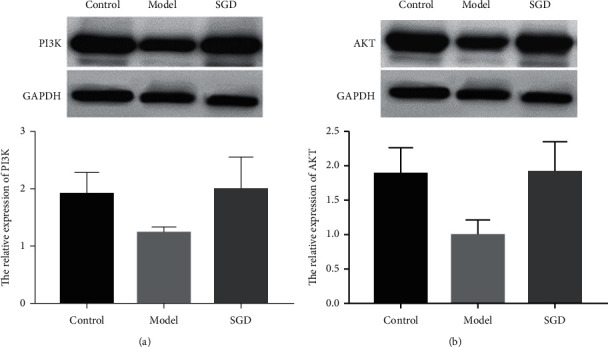
Effects of Shaoyao Gancao decoction (SGD) on PI3K (a) and AKT (b) protein expression.

The PI3K protein level of mice in the model group was 1.24 ± 0.11, which was lower than that of mice in the control group, 1.90 ± 0.38 (*P*=0.029). The PI3K protein level of mice in the SGD group was 1.98 ± 0.57, which was higher than that of the model group (*P*=0.021). The PI3K protein levels of mice in the control and SGD groups were similar, and no clear statistical difference was observed (*P*=0.773). The results are shown in Figure 8.

## 4. Discussion

Chinese medicine holds that the main causes of AA are liver and kidney deficiency, along with a deficiency of “essence” and blood. In addition, AA is also associated with endogenous wind produced by heated blood, liver stagnation, blood stasis, splenic deficiency, and blood weakness. Modern medical research has found that psychosomatic factors play an important role in the pathogenesis of AA [7], which coincides with the theory of liver stagnation and spleen deficiency in TCM. “Hair is the remainder of blood,” and blood is made of the essence of water and food, according to TCM. Although the spleen is the source of *qi* and blood, the liver is the master of draining and regulating *qi*, and the function of blood in the human body depends on the smooth regulation of *qi*. If one is worried, irritated, or overworked, the liver will become depressed and the spleen will be unable to transport and transform. Therefore, there is not enough *qi* and blood so that they cannot moisten the hair root and the hair falls off in pieces. SGD is often used clinically in the treatment of inflammatory and painful diseases. Relevant clinical studies [8] have shown that total glucosides of paeony capsules (TGPC) and compound glycyrrhizin tablets (CGT) containing Shaoyao or Gancao components are safe and effective in the treatment of AA alone or in combination. Paeoniflorin and glycyrrhetinic acid are the main active components in these two Chinese patent medicines, which have anti-inflammatory, immunomodulatory, and antianxiety effects. However, most of the relevant studies have focused on the active ingredients of single herbs, and there is a lack of network pharmacological studies on SGD.

According to the H-C-T network, the active components of SGD with high values include quercetin, kaempferol, 7-methoxy-2-methyl isoflavone, beta-sitosterol, and formononetin. Quercetin has anti-inflammatory, antiviral, antitumor, hypoglycemic, and immunomodulatory effects [9]. It was found that quercetin significantly inhibited the production of IL-6, MCP-1, IP-10, RANTES, GM-CSF, G-CSF, TNF-*α*, LIF, LIX, and VEGF [10], reduced proinflammatory cytokines (IL-1*β* and IL-6), and increased anti-inflammatory cytokines (IL-4, IL-10, and transforming growth factor-*β* 1) [11], thus exerting anti-inflammatory effects. Quercetin can exert immunomodulatory effects by inhibiting lymphocyte activation and proliferation [8]. Kaempferol has potent anti-inflammatory properties and has been found to inhibit inflammation-associated signaling pathways and suppress the release of inflammation-related factors, such as mitogen-activated protein kinases (MAPK), protein kinase C (PKC), phosphoinositide 3 kinase C (PKC), phosphoinositide 3-kinases (PI3K), and Janus kinase (JAK)/signal transducer and activator of transcription (STAT), thus exerting an anti-inflammatory effect [12]. In addition, kaempferol significantly inhibits the early activation of T lymphocytes and suppresses the proliferation of ConA-stimulated T cells, which have immunomodulatory effects [13]. *β*-sitosterol has biological activities such as anti-inflammatory, antioxidant, antitumor, antibacterial, antidepressant, and antihair loss [14]. Liao et al. [15] found that *β*-sitosterol can inhibit the activation of NLRP3, the production of CAS1, and the activation of the MAPK signaling pathway, leading to a significant decrease in cellular TNF-*α*, IL-1*β*, IL-6, and IL-8. In terms of immunomodulation, Alappatl and Valerio [16] showed that the combination of *β*-sitosterol and vitamin D3 can enhance the immune effects of macrophages. As an antidepressant, *β*-sitosterol can reduce the symptoms of depression by increasing norepinephrine, 5-serotonin, and its metabolite 5-hydroxyindole acetic acid in the brains of mice [17]. Formononetin can improve intrinsic immune function, inhibit apoptosis, and increase the conversion rate of splenocytes in mice [18]. For paeoniflorin, the main active ingredient of Shaoyao, experiments in various animal models have found that it may improve depression-like behavior and cellular damage caused by neurotoxicity associated with depression through mechanisms such as increasing monoamine neurotransmitters, improving hypothalamic-pituitary-adrenal axis activation, and attenuating neuroinflammatory factor damage and antiapoptotic cell death [19]. All the studies mentioned previously suggest that the main active components of SGD have anti-inflammatory, immunomodulatory, and anti-anxiety-depression effects, and all of these play an important role in the treatment of AA, reflecting its multicomponent synergistic effects.

Based on the results of the PPI network diagram analysis, it is evident that targets such as IL-6, PTGS2, TNF, VEGFA, CCL2, IL-1B, CXCL8, CASP3, MPO, and IL-10 are potential key targets of SGD in the treatment of AA. Proinflammatory factors such as IL-6 and IL-1B are essential for the differentiation of Th1 and Th17, which in turn affect the development of AA by influencing Th1 and Th17 cells involved in the development of AA [20]. Prostaglandin endoperoxide synthase 2 (PTGS2), also known as cyclooxygenase-2, is a proinflammatory response-inducing enzyme that plays an important role in pain and inflammatory mechanisms. TNF-*α* is a potent inhibitor of proliferation. TNF-*α*, IL-1*α*, and IL-1*β* were found to inhibit the growth of cultured hair follicles in vitro, leading to the pathogenesis of AA through activation of T cells [21]. Hair follicle immune privilege (HF IP) is an important link in the pathogenesis of AA, and related triggers can cause the release of IFN-*γ* and TNF-*α*, leading abnormal expression of MHC class I molecules in hair follicles during the anagen phase and disrupting HF IP, thus leading to the pathogenesis of AA. IL-10 is a Th2 cytokine that inhibits the synthesis of cytokines such as IFN-*γ*, IL-2, and TNF-*α* [22]. It was found that increased secretion of IL-10 could inhibit the expression of MHC-I and MHC-II, the action of IFN-*γ*, and promote the re-establishment of HF IP, which facilitates hair regrowth [23]. CCL2 is a member of the CC subfamily of chemokines, which recruits inflammatory cells to the site of the lesion and induces the synthesis of cytokines such as IL-2 and IL-6, thus exerting inflammatory suppressive and immunomodulatory effects. Elevated levels of Th1-related markers such as CCL2 have been found in patients with long-term AA [24], so it may be involved in the pathogenesis of AA by participating in the imbalance of Th1/Th2 cell subsets.

The results of the GO analysis showed that the biological processes involved in AA treatment with SGD were mainly inflammatory response, immune response, oxidation-reduction process, etc. Previous studies showed that SGD had anti-inflammatory and immunomodulatory effects, inhibiting the production of prostaglandin E2 (PGE2), nitric oxide (NO), and interleukin-6 (IL-6) [25], as well as regulating the ratio of CD4+CD25+Foxp3+ regulatory T cells [26]. Meanwhile, IL-6 was shown to be involved in AA pathogenesis as a proinflammatory factor; PGE2 inhibited macrophage function, reduced Thl cell proliferation and IL-1 and TNF-*α* synthesis, increased IL-4 production, and downregulated the effects of type I cytokines in treating AA by modulating immunity and anti-inflammation [27].

In the KEGG enrichment analysis of target genes, the PI3K-AKT signaling pathway, osteoclast differentiation, hepatitis B, and Jak-STAT signaling pathways were enriched to a higher degree and were closely related to AA. It was found that many cytokines involved in the pathogenesis of autoimmune and inflammatory diseases transmit intracellular signals through the JAK/STAT signaling pathway; for example, IL-6 can bind to JAK1 to exert biological effects [28]. Meanwhile, biopsies of AA skin lesions showed JAK3 overexpression and mildly elevated JAK1 and JAK2 expression, suggesting an important role for the JAK/STAT pathway in the pathogenesis of AA [29]. The emergence of JAK inhibitors in recent years has been a breakthrough in the treatment of AA. Although JAK inhibitors have not yet been approved by the U.S. Food and Drug Administration for the treatment of AA, several clinical studies have shown that JAK inhibitors such as tofacitinib can effectively treat AA [30–32]. The PI3K-AKT signaling pathway is widely present in cells and is involved in the regulation of cell growth, proliferation, and differentiation [33]. Current studies have focused on its relationship with the development, progression, treatment, and regression of various types of malignancies. Wachstein et al. [34] found that specific inhibitors of PI3K/AKT reduced the immunosuppressive function of HSP-70-treated T-regs cells by increasing the secretion of IFN-*γ*, TNF-*α*, IL-10, and TGF-*β*. Studies of AA patients [35] suggest that Th17/Treg cell imbalance and related cytokines are involved in the development of AA. This leads to the speculation that the PI3K-AKT signaling pathway may play a role in AA pathogenesis through immunomodulatory, anti-inflammatory, and antiapoptotic effects. However, the pathways of osteoclast differentiation and hepatitis B, which are at the top of the enrichment index, have not been reported in the literature to be associated with AA pathogenesis.

The molecular docking results showed that the binding energy of the main active components of SGD to the core protein was less than −5 kcal/mol, among which quercetin had the best binding property to PTGS2 (−9.2 kcal/mol), thus verifying the effect of SGD for the treatment of AA at the molecular level.

Through animal experiments, we initially verified the possible mechanism of SGD in the treatment of AA. First, unlike previous studies that reported upregulation of antiapoptotic protein p-Akt expression in lymph node cells of AA mice [36], this study found that PI3K and AKT protein levels were significantly lower in the AA model group compared with the control group of mice, suggesting a significant correlation between the onset of AA and the PI3K/AKT pathway. In addition, by comparing the PI3K and AKT protein levels between the AA model group and the SGD group, we found that SGD could effectively upregulate the expression of these two proteins; thus it was hypothesized that SGD could affect the secretion of related factors and improve cellular immune function through the PI3K/AKT pathway.

## 5. Conclusions

Utilizing network pharmacology's multilevel (drug-component-target-pathway) characteristics, this study determined the features of SGD responsible for drug synergy and the holistic concept of TCM and successfully predicted possible pharmacological mechanisms of SGD for the treatment of AA. Furthermore, the potential key pathway, the PI3K-AKT signaling pathway, was verified by animal experiments, providing a basis for exploring mechanisms of SGD in AA treatment.

In this study, network pharmacology findings suggested that the possible mechanism of SGD in AA treatment is through acting on IL-6, PTGS2, TNF, CCL2, IL-1B, IL-10, and other targets, mainly regulating the PI3K-AKT signaling pathway and Jak-STAT signaling pathway, thus exerting anti-inflammatory, immunomodulatory, and antidepressant functions. However, network pharmacology cannot predict the specific regulation of drugs on the targets, and false-positives may occur, so further experiments are needed to verify the results. Our subsequent animal experimental results showed that SGD could effectively upregulate PI3K and AKT protein expression in AA mice, which strongly supports the prediction of network pharmacology.

However, network pharmacology is based on predicting the main active components of traditional Chinese medicine, as a result of which we cannot analyze products of traditional Chinese medicine after absorption in the body. In addition, the experiment in this study was validated at the protein level, and subsequent further studies can investigate the changes in factors related to the PI3K/AKT pathway affected by SGD at the molecular level to explore the possible mechanism in greater depth.

## Figures and Tables

**Table 1:** Basic information on Shaoyao Gancao decoction (SGD) components.

Mol. ID	Compound ingredient	OB	DL	Herb
MOL001910	11-Alpha,12-alpha-epoxy-3-beta-23-dihydroxy-30-norolean-20-en-28,12-beta-olide	64.77	0.38	Shaoyao
MOL001918	Paeoniflorgenone	87.59	0.37	Shaoyao
MOL001919	(3S,5R,8R,9R,10S,14S)-3,17-dihydroxy-4,4,8,10,14-pentamethyl-2,3,5,6,7,9-hexahydro-1H-cyclopenta[a]phenanthrene-15,16-dione	43.56	0.53	Shaoyao
MOL001921	Lactiflorin	49.12	0.8	Shaoyao
MOL001924	Paeoniflorin	53.87	0.79	Shaoyao
MOL001925	Paeoniflorin_qt	68.18	0.4	Shaoyao
MOL001928	Albiflorin_qt	66.64	0.33	Shaoyao
MOL001930	Benzoyl paeoniflorin	31.27	0.75	Shaoyao
MOL000211	Mairin	55.38	0.78	Shaoyao,Gancao
MOL000358	Beta-sitosterol	36.91	0.75	Shaoyao
MOL000359	Sitosterol	36.91	0.75	Shaoyao,Gancao
MOL000422	Kaempferol	41.88	0.24	Shaoyao,Gancao
MOL000492	(+)-Catechin	54.83	0.24	Shaoyao
MOL001484	Inermine	75.18	0.54	Gancao
MOL001792	DFV	32.76	0.18	Gancao
MOL002311	Glycyrol	90.78	0.67	Gancao
MOL000239	Jaranol	50.83	0.29	Gancao
MOL002565	Medicarpin	49.22	0.34	Gancao
MOL000354	Isorhamnetin	49.6	0.31	Gancao
MOL003656	Lupiwighteone	51.64	0.37	Gancao
MOL003896	7-Methoxy-2-methyl isoflavone	42.56	0.2	Gancao
MOL000392	Formononetin	69.67	0.21	Gancao
MOL000417	Calycosin	47.75	0.24	Gancao
MOL004328	Naringenin	59.29	0.21	Gancao
MOL004805	(2S)-2-[4-Hydroxy-3-(3-methylbut-2-enyl)phenyl]-8,8-dimethyl-2,3-dihydropyrano[2,3-f]chromen-4-one	31.79	0.72	Gancao
MOL004806	Euchrenone	30.29	0.57	Gancao
MOL004808	Glyasperin B	65.22	0.44	Gancao
MOL004810	Glyasperin F	75.84	0.54	Gancao
MOL004811	Glyasperin C	45.56	0.4	Gancao
MOL004814	Isotrifoliol	31.94	0.42	Gancao
MOL004815	(E)-1-(2,4-Dihydroxyphenyl)-3-(2,2-dimethylchromen-6-yl)prop-2-en-1-one	39.62	0.35	Gancao
MOL004820	Kanzonols W	50.48	0.52	Gancao
MOL004824	(2S)-6-(2,4-Dihydroxyphenyl)-2-(2-hydroxypropan-2-yl)-4-methoxy-2,3-dihydrofuro[3,2-g]chromen-7-one	60.25	0.63	Gancao
MOL004827	Semilicoisoflavone B	48.78	0.55	Gancao
MOL004828	Glepidotin A	44.72	0.35	Gancao
MOL004829	Glepidotin B	64.46	0.34	Gancao
MOL004833	Phaseolinisoflavan	32.01	0.45	Gancao
MOL004835	Glypallichalcone	61.6	0.19	Gancao
MOL004838	8-(6-Hydroxy-2-benzofuranyl)-2,2-dimethyl-5-chromenol	58.44	0.38	Gancao
MOL004841	Licochalcone B	76.76	0.19	Gancao
MOL004848	Licochalcone G	49.25	0.32	Gancao
MOL004849	3-(2,4-Dihydroxyphenyl)-8-(1,1-dimethylprop-2-enyl)-7-hydroxy-5-methoxy-coumarin	59.62	0.43	Gancao
MOL004855	Licoricone	63.58	0.47	Gancao
MOL004856	Gancaonin A	51.08	0.4	Gancao
MOL004857	Gancaonin B	48.79	0.45	Gancao
MOL004860	Licorice glycoside E	32.89	0.27	Gancao
MOL004863	3-(3,4-Dihydroxyphenyl)-5,7-dihydroxy-8-(3-methylbut-2-enyl)chromone	66.37	0.41	Gancao
MOL004864	5,7-Dihydroxy-3-(4-methoxyphenyl)-8-(3-methylbut-2-enyl)chromone	30.49	0.41	Gancao
MOL004866	2-(3,4-Dihydroxyphenyl)-5,7-dihydroxy-6-(3-methylbut-2-enyl)chromone	44.15	0.41	Gancao
MOL004879	Glycyrin	52.61	0.47	Gancao
MOL004882	Licocoumarone	33.21	0.36	Gancao
MOL004883	Licoisoflavone	41.61	0.42	Gancao
MOL004884	Licoisoflavone B	38.93	0.55	Gancao
MOL004885	Licoisoflavanone	52.47	0.54	Gancao
MOL004891	Shinpterocarpin	80.3	0.73	Gancao
MOL004898	(E)-3-[3,4-Dihydroxy-5-(3-methylbut-2-enyl)phenyl]-1-(2,4-dihydroxyphenyl)	46.27	0.31	Gancao
	prop-2-en-1-one			
MOL004903	Liquiritin	65.69	0.74	Gancao
MOL004904	Licopyranocoumarin	80.36	0.65	Gancao
MOL004905	3,22-Dihydroxy-11-oxo-delta(12)-oleanene-27-alpha-methoxycarbonyl-29-oic acid	34.32	0.55	Gancao
MOL004907	Glyzaglabrin	61.07	0.35	Gancao
MOL004908	Glabridin	53.25	0.47	Gancao
MOL004910	Glabranin	52.9	0.31	Gancao
MOL004911	Glabrene	46.27	0.44	Gancao
MOL004912	Glabrone	52.51	0.5	Gancao
MOL004913	1,3-Dihydroxy-9-methoxy-6-benzofurano[3,2-c]chromenone	48.14	0.43	Gancao
MOL004914	1,3-Dihydroxy-8,9-dimethoxy-6-benzofurano[3,2-c]chromenone	62.9	0.53	Gancao
MOL004915	Eurycarpin A	43.28	0.37	Gancao
MOL004917	Glycyroside	37.25	0.79	Gancao
MOL004924	(-)-Medicocarpin	40.99	0.95	Gancao
MOL004935	Sigmoidin-B	34.88	0.41	Gancao
MOL004941	(2R)-7-Hydroxy-2-(4-hydroxyphenyl) chroman-4-one	71.12	0.18	Gancao
MOL004945	(2S)-7-Hydroxy-2-(4-hydroxyphenyl)-8-(3-methylbut-2-enyl)chroman-4-one	36.57	0.32	Gancao
MOL004948	Isoglycyrol	44.7	0.84	Gancao
MOL004949	Isolicoflavonol	45.17	0.42	Gancao
MOL004957	HMO	38.37	0.21	Gancao
MOL004959	1-Methoxyphaseollidin	69.98	0.64	Gancao
MOL004961	Quercetin der.	46.45	0.33	Gancao
MOL004966	3′-Hydroxy-4′-O-methylglabridin	43.71	0.57	Gancao
MOL000497	Licochalcone a	40.79	0.29	Gancao
MOL004974	3′-Methoxyglabridin	46.16	0.57	Gancao
MOL004978	2-[(3R)-8,8-Dimethyl-3,4-dihydro-2H-	36.21	0.52	Gancao
	pyrano[6,5-f]chromen-3-yl]-5-			
	methoxyphenol			
MOL004980	Inflacoumarin A	39.71	0.33	Gancao
MOL004985	Icos-5-enoic acid	30.7	0.2	Gancao
MOL004988	Kanzonol F	32.47	0.89	Gancao
MOL004989	6-Prenylated eriodictyol	39.22	0.41	Gancao
MOL004990	7,2′,4′-Trihydroxy-5-methoxy-3-	83.71	0.27	Gancao
	arylcoumarin			
MOL004991	7-Acetoxy-2-methylisoflavone	38.92	0.26	Gancao
MOL004993	8-Prenylated eriodictyol	53.79	0.4	Gancao
MOL004996	Gadelaidic acid	30.7	0.2	Gancao
MOL000500	Vestitol	74.66	0.21	Gancao
MOL005000	Gancaonin G	60.44	0.39	Gancao
MOL005001	Gancaonin H	50.1	0.78	Gancao
MOL005003	Licoagrocarpin	58.81	0.58	Gancao
MOL005007	Glyasperins M	72.67	0.59	Gancao
MOL005008	Glycyrrhiza flavonol A	41.28	0.6	Gancao
MOL005012	Licoagroisoflavone	57.28	0.49	Gancao
MOL005013	18*α*-Hydroxyglycyrrhetic acid	41.16	0.71	Gancao
MOL005016	Odoratin	49.95	0.3	Gancao
MOL005017	Phaseol	78.77	0.58	Gancao
MOL005018	Xambioona	54.85	0.87	Gancao
MOL005020	Dehydroglyasperins C	53.82	0.37	Gancao
MOL000098	Quercetin	46.43	0.28	Gancao

**Table 2:** Details of the 45 potential core targets.

Number	Target	Degree
1	IL-6	38
2	TNF	35
3	PTGS2	35
4	VEGFA	34
5	CCL2	33
6	CXCL8	32
7	IL-1B	32
8	IL-10	31
9	MPO	31
10	CASP3	31
11	EGF	30
12	IL-4	29
13	VCAM1	28
14	ICAM1	28
15	NOS3	27
16	IFNG	27
17	NOS2	26
18	SELE	26
19	PPARG	26
20	CRP	26
21	CXCL10	25
22	IL-2	25
23	SERPINE1	25
24	SPP1	22
25	CD40LG	22
26	IL-1A	21
27	STAT1	21
28	CXCL2	20
29	NFE2L2	17
30	THBD	16
31	SOD1	15
32	PTGS1	14
33	PON1	11
34	GJA1	10
35	CYP1A1	10
36	CYP3A4	9
37	RXRA	7
38	NR1I2	7
39	CYP1B1	7
40	ODC1	6
41	XDH	5
42	AKR1C1	4
43	ERBB3	3
44	LTA4H	2
45	BIRC5	1

## Data Availability

The data are available from the Universal Protein Database (UniProt, https://www.uniprot.org/), the Drugbank database (https://www.drugbank.ca/), the GeneCards database (https://www.genecards.org; version 4.11.0), the Online Mendelian Inheritance in Man (OMIM) (https://omim.org/), and the Disgenet database (https://www.disgenet.org/).
